# Functional variants at the 21q22.3 locus involved in breast cancer progression identified by screening of genome-wide estrogen response elements

**DOI:** 10.1186/s13058-014-0455-1

**Published:** 2014-10-09

**Authors:** Chia-Ni Hsiung, Hou-Wei Chu, Yuan-Ling Huang, Wen-Cheng Chou, Ling-Yueh Hu, Huan-Ming Hsu, Pei-Ei Wu, Ming-Feng Hou, Jyh-Cherng Yu, Chen-Yang Shen

**Affiliations:** 1 Institute of Biomedical Sciences, Academia Sinica, Academy Street, Taipei, 115 Taiwan; 2 Taiwan Biobank, Academia Sinica, Academy Street, Taipei, 115 Taiwan; 3 Department of Surgery, National Defense Medical College, Tri-Service General Hospital, Chenggong Road, Taipei, 114 Taiwan; 4 Cancer Center and Department of Surgery, Kaohsiung Medical University Chung-Ho Memorial Hospital, Tzyou 1st Road, Kaohsiung, 804 Taiwan; 5 College of Public Health, China Medical University, Hsueh-Shih Road, Taichung, 404 Taiwan

## Abstract

**Introduction:**

Estrogen forms a complex with the estrogen receptor (ER) that binds to estrogen response elements (EREs) in the regulatory region of estrogen-responsive genes and regulates their transcription. Sequence variants in the regulatory regions have the potential to affect the transcription factor–regulatory sequence interaction, resulting in altered expression of target genes. This study explored the association between single-nucleotide polymorphisms (SNPs) within the ERE-associated sequences and breast cancer progression.

**Methods:**

The ERE-associated sequences throughout the whole genome that have been demonstrated to bind ERα *in vivo* were blasted against online information from SNP data sets and 54 SNPs located adjacent to estrogen-responsive genes were selected for genotyping in two independent cohorts of breast cancer patients: 779 patients in the initial screening stage and another 888 in the validation stage. Deaths due to breast cancer or recurrence of breast cancer were defined as the respective events of interest, and the hazard ratios of individual SNPs were estimated based on the Cox proportional hazards model. Furthermore, functional assays were performed, and information from publicly available genomic data and bioinformatics platforms were used to provide additional evidence for the associations identified in the association analyses.

**Results:**

The SNPs at 21q22.3 ERE were significantly associated with overall survival and disease-free survival of patients. Furthermore, these 21q22.3 SNPs (rs2839494 and rs1078272) could affect the binding of this ERE-associated sequence to ERα or Rad21 (an ERα coactivator), respectively, which resulted in a difference in ERα-activated expression of the reporter gene.

**Conclusion:**

These findings support the idea that functional variants in the ERα-regulating sequence at 21q22.3 are important in determining breast cancer progression.

**Electronic supplementary material:**

The online version of this article (doi:10.1186/s13058-014-0455-1) contains supplementary material, which is available to authorized users.

## Introduction

The roles of estrogen receptor α (ERα) in initiating tumor development in breast cancer, regulating progression and determining therapeutic protocols and efficacy are well documented [1],[2]. However, not all patients with the same ERα status manifest the same cancer progression or response to hormone therapy, and individual variations in breast cancer progression have remained an issue of particular concern. Although ERα can be activated in an estrogen-independent manner, the classical activation mechanism involves the binding of ERα to estrogen and other coactivator proteins to form the estrogen-bound ER complex, which functions as a transcriptional regulator [3],[4]. The DNA-binding domain of ERα binds to estrogen response elements (EREs) in the regulatory region of estrogen-responsive genes, activating or repressing their transcription and consequently mediating physiological or tumorigenic effects. Since sequence variants, such as single-nucleotide polymorphisms (SNPs), located in the regulatory regions of genes have the potential to affect protein (transcription factor)–DNA (regulatory region) interactions, resulting in altered expression of target genes [5],[6]. We previously examined the hypothesis that genetic variations of genome-wide EREs might be associated with breast cancer development, and we identified a significant effect of several ERE-associated SNPs on breast cancer risk [7]. However, because the ERE sites we examined were based on prediction by a computational algorithm and lacked confirmation by results from cell-based assays, it was not possible to know whether such EREs indeed function as predicted *in vivo*. In the present study, we explored the association between genetic variants within these ERE-associated sequences and breast cancer progression. Importantly, these ERE-associated sequences scattered throughout the whole genome have been shown, by using chromatin immunoprecipitation (ChIP)–based methods, to bind ERα *in vivo*[8]-[10]. This is a promising approach for identifying the breast tumorigenic contribution of EREs on a genome-wide scale. Furthermore, we performed functional assays and used information from publicly available genomic data and bioinformatics platforms to provide additional evidence for the association identified in the association analysis. The results obtained by the combined use of these different approaches in this multistage study support the idea that functional variants in the ERα-regulating sequence at 21q22.3 are important in determining breast cancer progression.

## Methods

### Study participants

**Figure 1:**
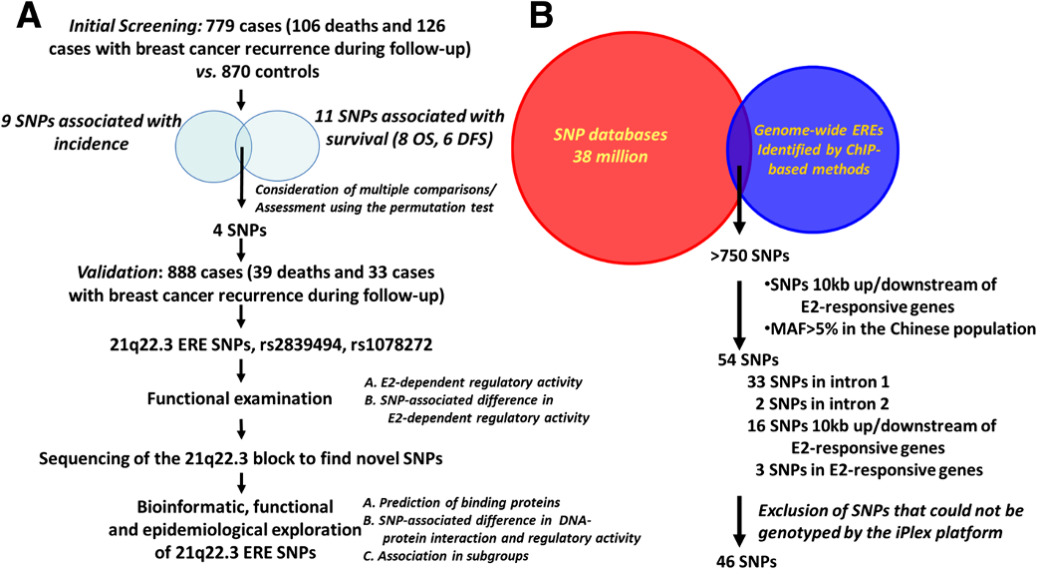
**Identification and functional examination of genome-wide estrogen response element–associated single-nucleotide polymorphisms associated with breast cancer progression. (A)** Flow diagram showing the sequential steps in the present study. **(B)** Selection of single-nucleotide polymorphisms (SNPs) to be genotyped. SNPs located within regions that bind estrogen receptor α (ERα) *in vivo* were selected, and then they were examined for whether they were located within the regions 10 kb 3' or 10 kb 5' of known estrogen (estradiol (E2))-responsive genes. As a result, after excluding those that could not be genotyped by using the iPLEX platform, 46 estrogen response element (ERE)–associated SNPs were genotyped. ChIP, Chromatin immunoprecipitation; DFS, Disease-free survival; OS, Overall survival.

Two independent cohorts of patients with incident primary breast cancer, 779 of whom were in the initial screening stage and another 888 in the validation stage (Figure 1A), were included in the present study. All of the patients were part of our ongoing cooperative study aimed at understanding the causes and progression of breast cancer in Taiwan. Their characteristics have been described in detail elsewhere [7],[11]-[13]. Two independent groups of women without a history of cancer were recruited. One of these groups comprised 870 women from the same source as the patients in the initial screening phase. That group was used as the control group to explore the association between SNPs and breast cancer development. The other group comprised 903 women chosen from the National Biobank of Taiwan [14]; we used their data to provide background information about haplotype block and linkage disequilibrium (LD) between SNPs in our population.

This study was approved by the ethics committee of the institutional review board of the Academia Sinica, Taiwan, and informed consent was obtained from all study participants before the collection of data by personal interview.

### Single-nucleotide polymorphisms and genotyping

Genome-wide EREs were detected by ChIP using anti-ERα antibodies in different ERα-positive breast cancer cells [8]-[10]. These ERE sites were blasted against the SNP database, resulting in the identification of the ERE-associated SNPs. Multiple steps were used to select the SNPs for genotyping; these steps are described in the Results section. In the initial screening stage, SNPs were genotyped in all samples tested using Sequenom iPLEX technology (Sequenom, Hamburg, Germany). Duplicate positive and negative controls were included on all plates, with genotypes autocalled by using specialized software (MassARRAY Typer version 3.4; Sequenom) and subsequently confirmed by visual assessment of the data. All assays were performed by individuals blinded to the case versus control status of the samples. As a quality control, we repeated the genotyping on 10% of the samples, and all genotype scoring was performed and checked separately by one reviewer who was unaware of the case versus control status. The concordance rate for replicate samples was 100%. In the validation stage, the genotyping data at specific SNPs for 888 patients with incident breast cancer were used. These SNPs showed strong LD with those significant SNPs identified in the initial screening stage. These 888 patients had been included in the international Collaborative Oncological Gene-environment Study (COGS), in which genotyping was performed using a customized Illumina Infinium BeadChip array (Illumina, San Diego, CA, USA) [15],[16]. Using the National Biobank of Taiwan, a total of 642,832 SNPs from 903 women were genotyped, and the details of the SNPs, how they were selected and the genotyping results are publicly available on the Taiwan Biobank website [14].

### Statistical analysis

To identify putative high-risk genotypes of ERE-associated SNPs for breast cancer incidence in the initial screening stage, we followed our previously established sequential steps [7],[11]-[13] to analyze the data. Hardy-Weinberg equilibrium was tested to ensure that the chance of genotyping error was small. Logistic regression was used to estimate the odds ratio of breast cancer associated with harboring an individual genotype. Data on ERα status (positive or negative), histologic grade (well-differentiated to poorly differentiated) and clinical stage (I to IV) were collected from hospital medical records. We were thereby able to determine whether an ERE-associated SNP influenced prognosis. To this end, we calculated the overall survival (OS) rate and the breast cancer–specific survival rate (that is, disease-free survival (DFS)) using the Kaplan-Meier method and the two-tailed logrank test, with death due to breast cancer or recurrence of breast cancer defined as the respective event of interest. In addition, the hazard ratios (HRs) of individual SNPs associated with OS or DFS were estimated based on the Cox proportional hazards model, considering the effect of the patients’ age, ER status and cancer stage. All statistical analyses were performed using SAS version 9.1 software (SAS Institute, Cary, NC, USA), and all tests were based on a two-sided probability. To address the issue of false-positivity due to multiple tests, a permutation test [17] was performed when needed.

LD plots of the D’ values for SNPs within the same haplotype block were produced using the Haploview program [18]. Haplotype estimation was performed on individuals for whom complete genotype data were available across all polymorphic sites, and the highest probability haplotypes, estimated using the expectation maximization algorithm of SAS Genetics 9.1 (SAS Institute), were assigned to each study participant [19].

### Sequencing

Sanger sequencing was performed to detect single-nucleotide variants within specific genomic regions.

### Cell culture

The human breast cancer cell line MCF-7 was purchased from the Taiwan Bioresource Collection and Research Center. The cells were cultured in Dulbecco’s modified Eagle’s medium (Sigma-Aldrich, St Louis, MO, USA), supplemented with 10% fetal bovine serum (Gibco, Grand Island, NY, USA).

### Construct generation, transfection and site-directed mutagenesis

The ERα-binding regions containing specific ERE-associated SNPs were amplified by PCR from human genomic DNA and inserted into the promoter region of the pGL3-Basic vector (Promega, Madison, WI, USA) at the NheI-XhoI restriction site. ERα- and Rad21-expressing constructs were amplified from cDNA. The former were cloned into the EcoRI-XhoI sites, and the latter into the NotI-XhoI sites, of the mammalian expression vector pcDNA3-Flag. For site-directed mutagenesis, specific point mutations (that is, variant alleles) were generated using QuikChange Site-Directed Mutagenesis kits (Stratagene/Agilent Technologies, Santa Clara, CA, USA) according to the manufacturer’s instructions. Transfection of plasmids was performed using Lipofectamine 2000 reagent (Invitrogen, Carlsbad, CA, USA) according to the manufacturer’s instructions.

### Luciferase reporter assay

The luciferase constructs and pRL-TK, which encodes Renilla luciferase, were cotransfected into 5 × 10^4^ MCF-7 cells in 24-well plates. After 48 hours, the cells were lysed in a single freeze–thaw cycle in passive lysis buffer. The lysate was then centrifuged at 12,000 *g* for 10 minutes at 4°C, and the luciferase activities in the supernatant were measured using Dual-Luciferase Reporter Assay System kits (Promega). The relative activity of luciferase was determined using the Renilla luciferase signal as the reference.

### Chromatin immunoprecipitation

ChIP was performed based on our previous protocol [20] using EZ-Magna ChIP G kits (EMD Millipore, Billerica, MA, USA) following the manufacturer’s instructions and using anti-Flag monoclonal antibody (F3165; Sigma-Aldrich) for the precipitation stage. The immunoprecipitate was eluted with 50 μl of the supplied Tris-ethylenediaminetetraacetic acid buffer, and 2 μl of DNA were used in quantitative PCR (qPCR). The primer pairs used for the ChIP PCR were 5'-CCGGCCATCTCTCACTATGAA-3' and 5'-CCTTCCCGCCAGGGTAAATAC-3' for *TFF1* and 5'-CTTGAGGTGCTTCGAGACAGTG-3' and 5'-CACCTGCTTCAAAGTGAGTGAG-3' for *21q22.3*.

## Results

### Characteristics of the patient cohorts

The risk profile of breast cancer in our study participants was similar to that found in our previous studies [11]-[13] and in other breast cancer studies [21]. Development of breast cancer was found to be highly associated with reproductive risk factors, including early menarche (adjusted odds ratio (aOR), 1.33; 95% confidence interval (CI), 1.09 to 1.64), nulliparity (aOR, 1.37; 95% CI, 1.03 to 1.85), low number of full-term pregnancies (less than two) (aOR, 1.12; 95% CI, 0.89 to 1.40) and no history of breastfeeding (aOR, 1.49; 95% CI, 1.21 to 1.83). Importantly, these significant associations between reproductive risk factors and breast cancer reveal the importance of the estrogen-related etiology of breast cancer in our participants, providing us with the opportunity to examine the contribution of EREs during breast tumorigenesis. Because the present study was focused on SNPs in ERE-associated sequences involved in determining breast cancer progression, we also examined factors in the clinical profile that correlated with OS and DFS. As expected, patient age at tumor onset, ER status and tumor stage were three major determinants in our cohort and were included in all of our analyses so that we could examine the effect of SNPs on breast cancer progression.

### Selection of estrogen response element–associated single-nucleotide polymorphisms for genotyping

Genome-wide ERα-binding sites have previously been detected using ChIP-based methods [8]-[10], and more than 1,500 binding regions have been identified. After blasting these regions against online information available from SNP data sets (UCSC Genome Browser, National Center for Biotechnology Information and HapMap databases), including more than 38 million SNPs throughout the whole genome, more than 750 SNPs were identified in ERα-associated sequences (Figure 1B). We next examined whether these SNPs were located adjacent to estrogen-responsive genes, as genetic variants near genes may affect the interactions of transcription factors with the promoter/enhancer/regulatory regions, resulting in altered mRNA expression. To do so, we used the bioinformatics tool GenePipe [22] to screen for SNPs located within the regions 10 kb 3' or 10 kb 5' of estrogen-responsive genes, identified by showing a significant change in expression when the ERα-positive breast cancer cells were treated with estrogen [8]-[10]. Taking statistical power considerations into account, we included only SNPs with a minor allele frequency greater than 5% in the Chinese population. As a result, 54 SNPs were identified, and, after excluding 8 that could not be genotyped in the iPLEX platform, a total of 46 SNPs were genotyped in all patients and controls in the initial screening stage (Figure 1A).

### Identification of single-nucleotide polymorphisms associated with breast cancer development and progression

**Figure 2:**
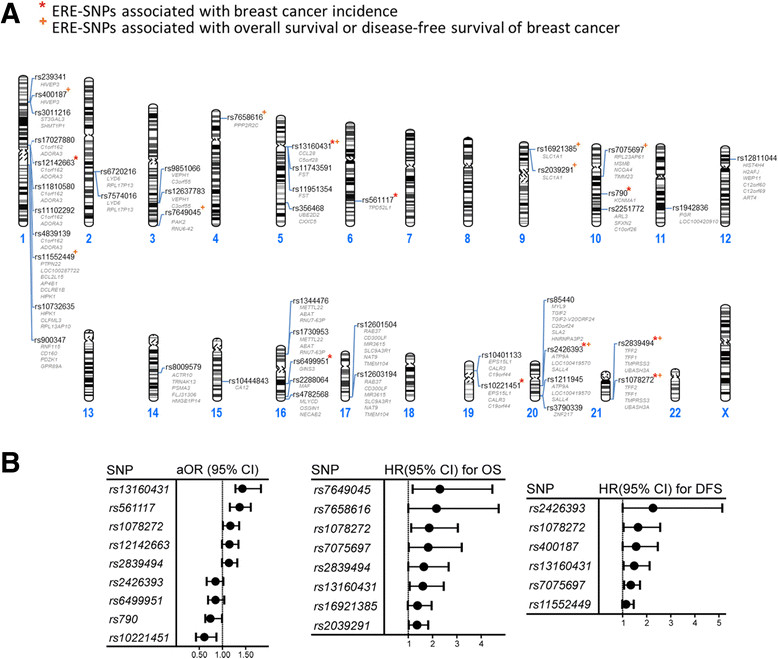
**Estrogen response element–associated single-nucleotide polymorphisms related to breast cancer incidence and progression.** Genomic sites **(A)** and adjusted odds ratios (aORs), hazard ratios (HRs) and corresponding 95% confidence intervals (CIs) **(B)** of the estrogen response element (ERE)–associated single-nucleotide polymorphisms (SNPs) found to be significantly associated with breast cancer incidence (left panel), overall survival (OS; center panel) or disease-free survival (DFS; right panel). Known genes adjacent to these SNPs are shown in gray in **(A)**. aORs were estimated by logistic regression analyses, and the HRs were estimated by applying the Cox proportional hazards model. In total, 779 patients (106 deaths and 126 cases with breast cancer recurrence during follow-up) and 870 controls were included in these analyses.

We next sought to determine the breast tumorigenic contribution of ERE-associated SNPs. In the initial screening stage, we examined whether the genotypic distribution of these 46 SNPs differed between cases and controls and between cases with different progression outcomes (that is, OS versus DFS) (Figure 1A). The frequencies of all SNPs in the controls agreed with those expected on the basis of the Hardy-Weinberg equilibrium, suggesting that genotyping errors were relatively unlikely. The results for the genotypic analysis showed that nine SNPs were associated with breast cancer incidence (Figure 2A and 2B, left panel) and that eleven SNPs were associated with survival (DFS or OS) (Figure 2A and 2B, center and right panels). Women carrying the homozygous variant genotype had a significantly increased aOR or HR (*P* <0.05) compared to women carrying the homozygous and heterozygous wild-type genotypes. The possibility of false-positives due to multiple testing is relatively unlikely, because the results of the permutation test [17], based on 10,000 random permutations, showed that these associations were significant (data not shown). Particular attention was focused on four SNPs, each of which was associated with both breast cancer incidence and progression (Figures 1A, 2A and 2B). Though the genetic variants associated with cancer progression are not necessarily those associated with cancer incidence, variants that play a dual role in different stages during tumorigenesis are certainly of more tumorigenic importance. Of these four SNPs, two (rs2839494 and rs1078272) are located in the same LD block at 21q22.3 and the other two at 5p12 and 20q13.2 (Figure 2A).

### Validation of 21q22.3 single-nucleotide polymorphisms for breast cancer progression

**Figure 3:**
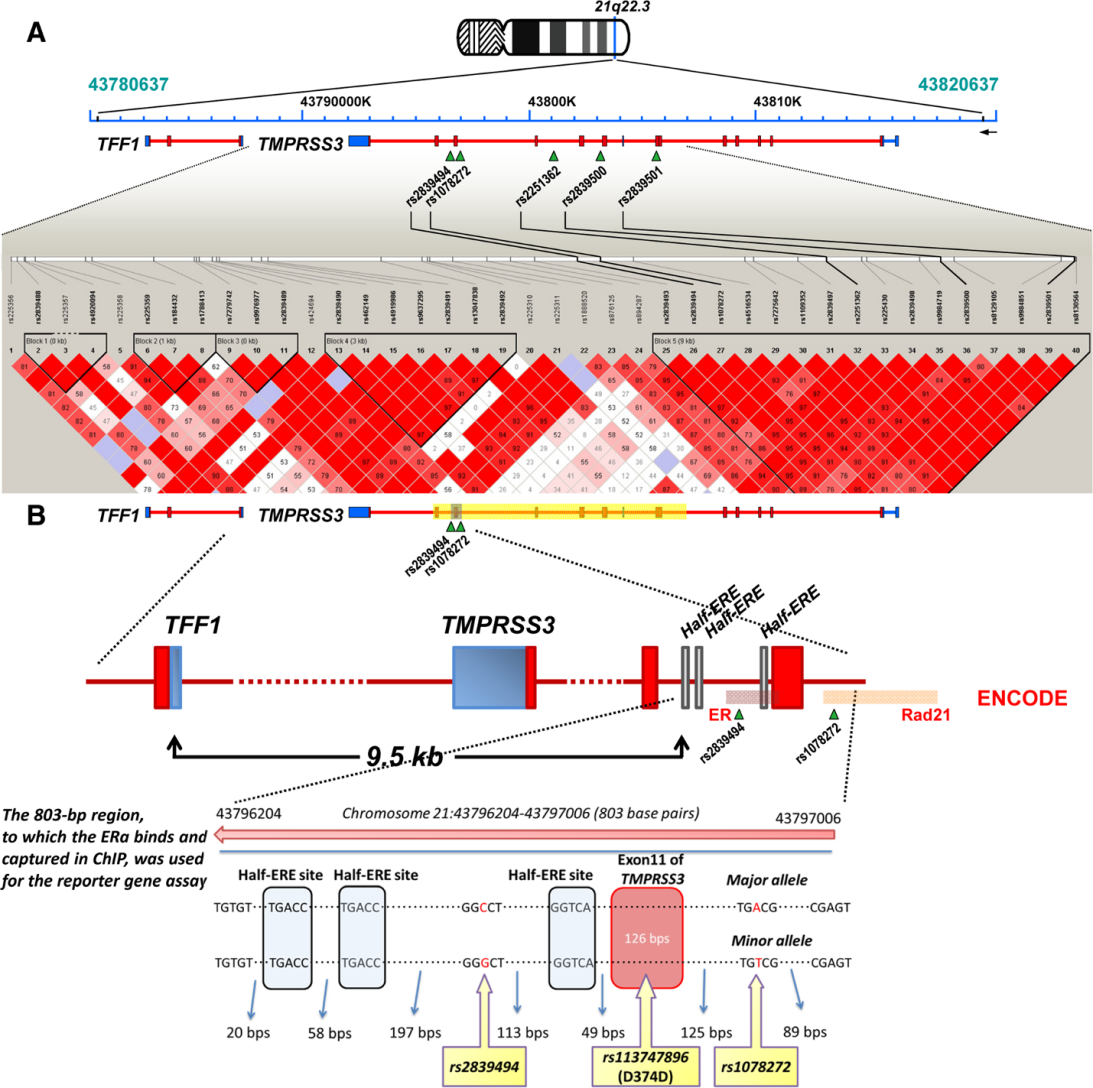
**Schematic diagrams of the 21q22.3 block and the 21q22.3 region that binds estrogen receptor α. (A)** Three single-nucleotide polymorphisms (SNPs) were found to be significantly associated with breast cancer progression, of which two (rs2839494 and rs1078272) were associated with estrogen response elements (EREs) and located at a region that binds estrogen receptor α (ERα). These two SNPs were used in the initial screening stage, and rs2251362 was used in the validation stage. Two additional SNPs, rs2839500 and rs2839501, were exonic SNPs detected by direct sequencing of the 21q22.3 block (see text for details). All five SNPs are located in the same 21q22.3 haplotype block. The linkage disequilibrium (LD) plot shows LD between the SNPs in 21q22.3 in Han Chinese women. The strength of the LD between SNPs, indicated by the color scheme, was measured using a combination of the statistic *D*' and the logarithm of odds (LOD) score (dark red shading, *D*' =1 and LOD score ≥2; light red shading, *D*' <1 and LOD score ≥2). **(B)** Top panel: The 21q22.3 block (yellow) and the 21q22.3 region to which ERα binds (gray). Middle panel: Enlarged view showing the half-ERE sites (thin gray bars), exons (red) and untranslated regions (blue) of *TMPRSS3/TFF1*, and the regions to which ERα and the ER coactivator Rad21 bind, detected by using the Encyclopedia of DNA Elements (ENCODE). Bottom panel: The 21q22.3 region to which ERα binds. The major and minor alleles of the three SNPs in this region are indicated. ChIP, Chromatin immunoprecipitation.

**Figure 4:**
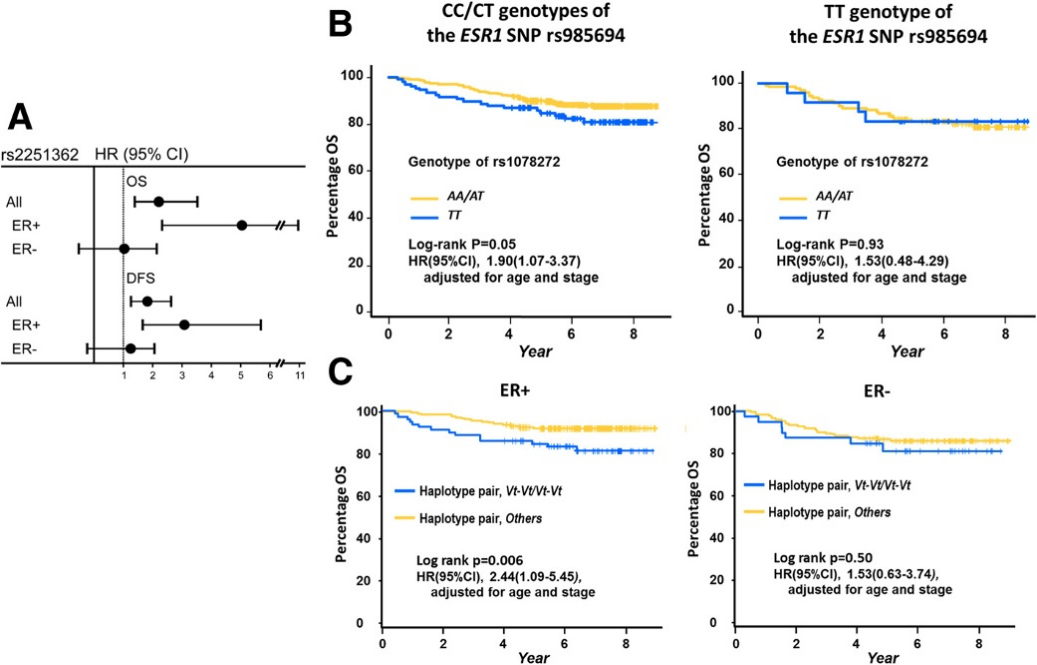
**Associations between single-nucleotide polymorphisms in 21q22.3 in tumors displaying different estrogen receptor status and breast cancer progression. (A)** The 21q22.3 single-nucleotide polymorphism (SNP) rs2251362 is significantly associated with both overall survival (OS) and disease-free survival (DFS) in all patients, especially in estrogen receptor–positive (ER+) patients, as detected in the validation stage. Hazard ratios (HRs) and 95% confidence intervals (95% CIs) were estimated based on the Cox proportional hazards model, considering the effects of the patients’ age, ER status and cancer stage. A total of 888 breast cancer patients (39 deaths and 33 patients with breast cancer recurrence during follow-up) were included. **(B)** The association between genetic polymorphism of the estrogen response element (ERE)–associated SNP in 21q22.3 (defined by rs1078272) and OS was significantly modified by the genotype of the SNP (that is, rs985694) of *ESR1*, the gene encoding the ER. **(C)** Association between genetic polymorphism of 21q22.3 detected by the haplotype pairs containing the two ERE-associated SNPs (rs2839494 and rs1078272) and OS of breast cancer patients with ER + tumors (left panel) or ER − tumors (right panel). The 779 patients (106 deaths and 126 patients with breast cancer recurrence during follow-up) in the initial screening were included in the analyses depicted in **(B)** and **(C)**. HRs and 95% CIs were estimated based on the Cox proportional hazards model, considering the effects of the patients’ age and cancer stage.

To confirm the significant associations between specific ERE-associated SNPs and breast cancer progression detected in the initial screening, we studied an independent cohort of 888 breast cancer patients (Figure 1A). The genotyping data for these patients were recently reported in the COGS genome-wide association study (GWAS) [15],[16]. We first validated that the SNPs at 5p12, 20q13.2 and 21q22.3, genotyped in our COGS patients, were in very strong LD (all LD coefficients between SNPs >0.95 in our population) with the four significant SNPs identified in our initial screening, not only in Han Chinese using HapMap data but also in Taiwanese women using Taiwan Biobank data [14]. These SNPs were then tested for their associations with cancer progression. The polymorphic status of 21q22.3, now reflected by rs2251362 (Figure 3A), remained significantly associated with OS and DFS in the validation stage (Figure 4A). More importantly, to examine whether an ERα-associated mechanism could explain this association, we stratified our patients based on the ER status of their tumors and found that the significant association between rs2251362 and cancer progression was present only in the ERα-positive patients (Figure 4A). These results prompted us to retrospectively check the interaction between 21q22.3 SNPs and the ER in the patients included in the initial screening. Consistent with the finding above, the association between breast cancer progression (indicated by OS) and ERE-associated SNP at 21q22.3 (that is, rs1078272) was more significant in patients harboring specific genotypes of a SNP (rs985694) of *ESR1*, the gene encoding ERα (Figure 4B). We also performed haplotype analysis in which a polymorphism of 21q22.3 was defined more precisely by the two ERE-associated SNPs rs2839494 and rs1078272. As shown in Figure 4C, women carrying the haplotype pair of the variant allele of both rs2839494 and rs1078272 (*Vt-Vt/Vt-Vt*) were found to manifest a significantly worse survival than those carrying other haplotype pairs, particularly in the ER-positive patients.

### Functional examination of the two estrogen response element–associated single-nucleotide polymorphisms at 21q22.3

**Figure 5:**
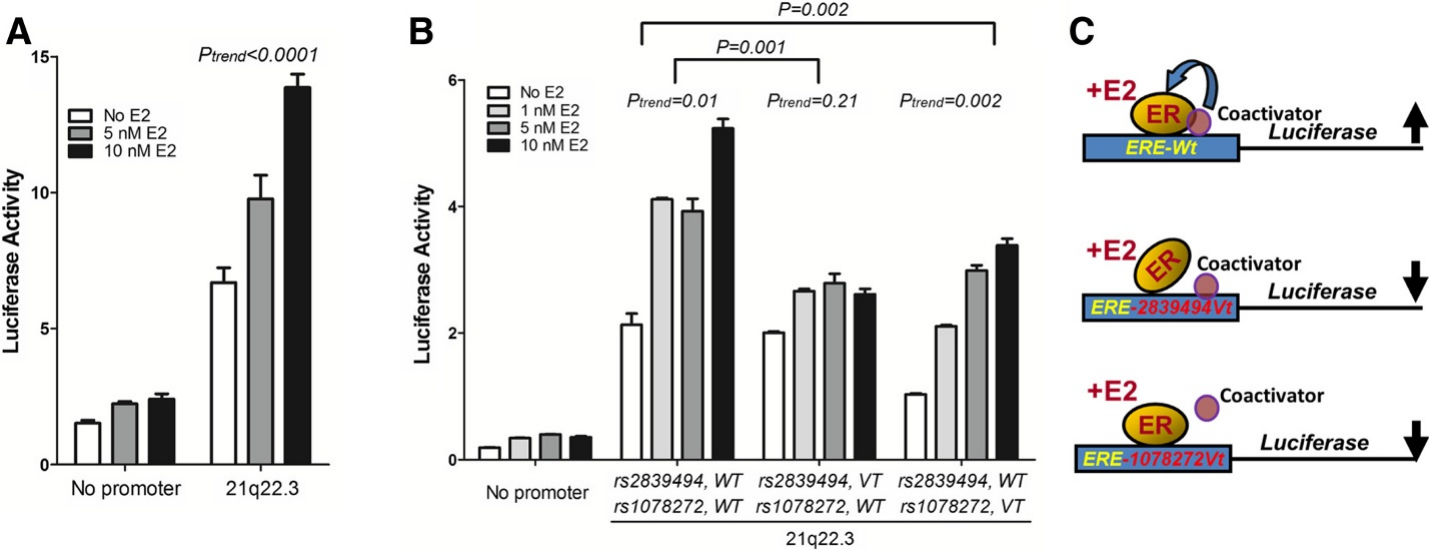
**Functional examination of the two estrogen response element–associated single-nucleotide polymorphisms rs2839494 and rs1078272 in 21q22.3.** **(A)** Activity in MCF-7 cells of the luciferase reporter gene, either alone (denoted as “No promoter”) or linked to the 21q22.3 region (that is, the segment shown in Figure 3B, bottom panel) to which estrogen receptor α (ERα) binds, and which contains the two estrogen response element (ERE)–associated single-nucleotide polymorphisms (SNPs) (denoted as “21q22.3”), in the presence or absence of estradiol (E2). **(B)** Decreased activity of the luciferase reporter gene in MCF-7 cells when linked to the 21q22.3 region harboring the variant allele (*VT*) of rs2839494 or rs1078272 compared to that containing both wild-type alleles (*WT*). The results shown in **(A)** and **(B)** are means ± standard deviations (*n* = 3). The *P*-values of differences between groups and the *P*-values for trends within groups were estimated by regression analysis. **(C)** Hypothetical model explaining the similar, but not identical, effects of the two ERE-associated SNPs at 21q22.3, showing that the variant allele of rs1078272 might block the binding of a coactivator to the 21q22.3 sequence (bottom panel), whereas the variant allele of rs2839494 might directly affect the binding of ERα (center panel), both decreasing reporter gene activity compared to the wild-type 21q22.3 sequence (top panel).

On the basis of the above-described results, functional studies were performed to examine the effect of these two ERE-associated SNPs at 21q22.3 (Figure 1A). As shown in Figure 3B, rs2839494 and rs1078272 are located in an ERα-binding segment spanning 803 bp, containing three half-ERE sequences and covering exon 10 of *TMPRSS3*. In addition, at a region 9.5 kb 5' of this segment, there is a gene, *TFF1*, which has been shown to be a suppressor of breast cancer in a mouse model [23]. We first demonstrated estradiol (E2)-dependent regulatory activity of this 803-bp segment by an E2 dose-dependent increase in reporter gene activity when the segment was linked to the luciferase reporter gene, the construct transfected into ER-positive MCF-7 cells and the transfected cells incubated with increasing levels of E2 (Figure 5A). To examine the effect of the variant alleles on transcription and to differentiate the effects of rs2839494 and rs1078272, we generated one variant allele of each SNP by mutating the wild type and tested their individual effects in regulating reporter gene activity. Each of the variant alleles led to significantly decreased luciferase activity (Figure 5B). However, interestingly, the two variant alleles resulted in different phenotypes with decreased ERα-regulated activity. The variant allele of rs2839494 completely abolished the response to E2, and the variant allele of rs1078272 decreased the response but maintained the dose–response relationship between E2 and reporter gene activity. This marked difference suggested different inhibition mechanisms (Figure 5C), which were examined in our following experiments.

### Direct sequencing to search for additional single-nucleotide polymorphisms within 21q22.3

To gain support for a causal role of these two ERE-associated SNPs (rs2839494 and rs1078272) in 21q22.3, we attempted to clarify alternative possibilities, including that (1) rs2839494 and rs1078272 might be in LD with an exon change in *TMPRSS3* that affects protein function, (2) other changes in this 21q22.3 block might be within regulatory sequences and affect the level of expression through transcriptional regulation and (3) alterations in other adjacent genes might increase susceptibility to breast cancer progression. Because *TMPRSS3* is the only gene in this 21q22.3 block (Figure 3A and 3B, top panel), the third possibility is unlikely. To examine the first possibility, using blood specimens from 100 healthy individuals, we performed exonic sequencing of all *TMPRSS3* exons within this block and identified three SNPs: rs2839500, rs2389501 and rs113747896 (Figure 3A and 3B, bottom panel; and Table 1). None of these SNPs are novel; the first two have been shown to have no pathogenic effect [24],[25], and the third, located within exon 10 of *TMPRSS3*, results in no amino acid change (D374D) (Figure 3B and Table 1). To examine the second possibility listed above, using blood specimens from 58 healthy individuals, we performed direct sequencing to identify any variants within this 21q22.3 block containing 9,940 bp (Table 1). We found that some of the identified SNPs were located at transcription factor binding sites, so we cannot totally exclude the possibility that these variants affect expression of E2/ERα-regulating genes. However, it is notable that the 803-bp ERE-associated sequence was the only segment within the 21q22.3 block that could bind ERα (Table 1). As a result, rs2839494 and rs1078272 appear to be candidate causal variants.

**Table 1:** **Direct sequencing of the 21q22.3 block to identify single-nucleotide variants** ^**a**^

	Base pairs	Half-ERE sites	SNPs^b^(***n***)	Novel SNPs^b^(***n***)	Exonic SNPs^c^(***n***)	Novel exonic SNPs^c^(***n***)	SNPs in the TF binding site^d^(***n***)	Binding to ERα detected by ChlP
21q22.3 block	9,940	17	37	6	3	0	10	Only the one below
ERα-binding sequence within 21q22.3	803	3	3	0	1	0	2	Yes

^a^ChIP, Chromatin immunoprecipitation; ERα, Estrogen receptor α; ERE, Estrogen response element; SNP, Single-nucleotide polymorphism; TF, Transcription factor; N, the number of nucleotide. ^b^Based on HapMap CHB (Han Chinese in Beijing, China), dbSNP137 and direct sequencing of blood specimens from 58 individuals. The novel SNPs were identified by the sequencing of 58 individuals. ^c^Based on HapMap CHB, dbSNP137 and exonic sequencing of blood specimens from 100 individuals, no exonic SNP was identified. ^d^Based on Encyclopedia of DNA Elements (ENCODE). The eight SNPs within the TF binding sites but outside the ERα-binding sequence showed no evidence of ERα binding.

### Bioinformatics and functional evidence that the Rad21-coactivated E2-induced increase in promoter activity is affected by ERE-associated SNPs in 21q22.3

It is notable that Encyclopedia of DNA Elements (ENCODE) data [26] show that rs2839494 and rs1078272 lie, respectively, within different binding regions of ERα and Rad21, a coactivator of ERα [27]. Given the difference in phenotype of the effect on E2-dependent regulatory activity between rs2839494 and rs1078272 (shown in Figure 5B), we speculated that these two SNPs might be involved in the protein–DNA interaction (that is, interaction of transcription factors with promoter/enhancer/regulatory regions) by different mechanisms (Figure 5C).

**Figure 6:**
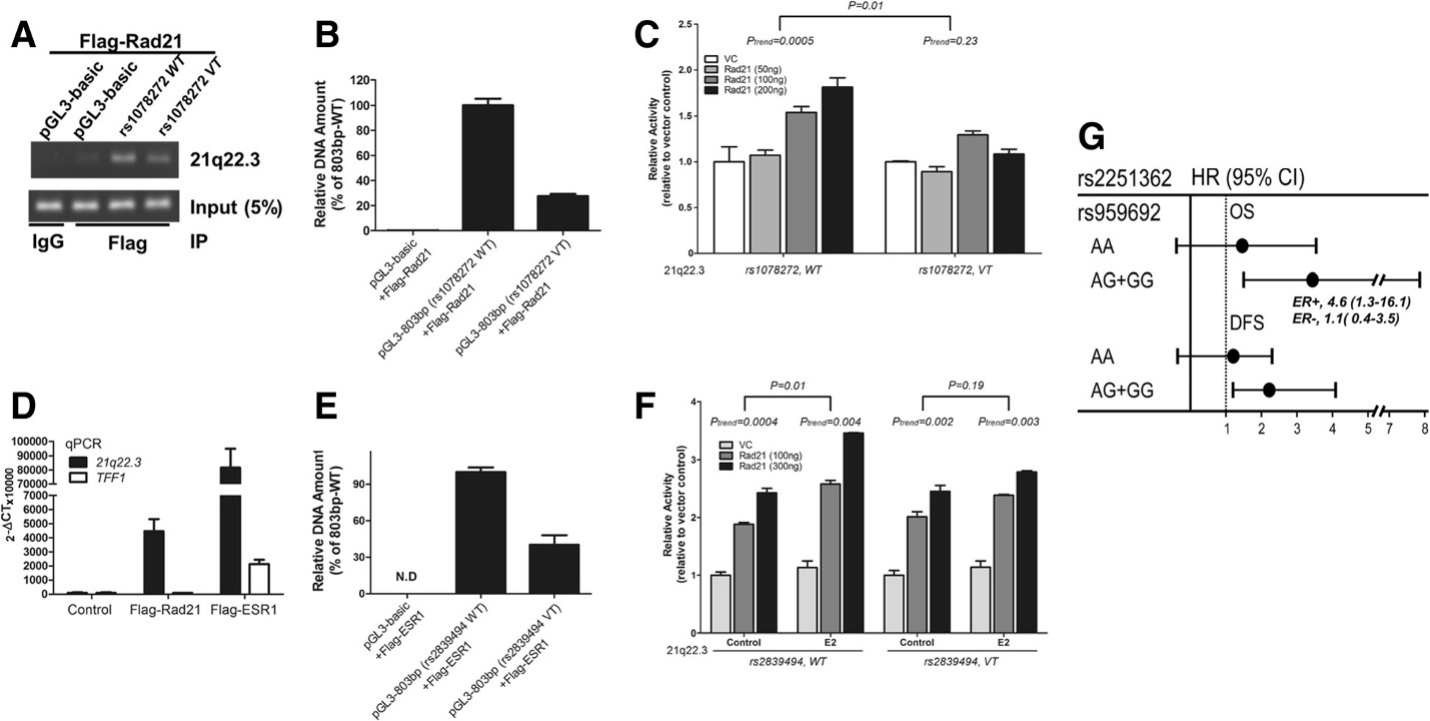
**Estrogen response element–associated single-nucleotide polymorphisms at 21q22.3 displaying similar, but not identical, effects on reporter gene activity regulated by estrogen receptor α, suggesting Rad21 coactivation of the estrogen receptor–regulated activity of the 21q22.3 fragment.** The variant allele of rs1078272 decreased Rad21 binding and Rad21 coactivation of estradiol (E2)-regulated reporter activity. Binding of the 21q22.3 fragment harboring the rs1078272 wild-type (*WT*) or variant allele (*VT*) to the transfected Flag-tagged Rad21 in MCF-7 cells was detected by chromatin immunoprecipitation (IP). **(A)** A typical result and **(B)** the real-time quantitative PCR (qPCR) results for the amount of 21q22.3 bound to Rad 21. IgG, Immunoglobulin G. **(C)** Regulatory activity of 21q22.3 on reporter in MCF-7 cells containing the rs1078272 wild-type or variant allele, transfected with vector control (VC) or different amounts (50, 100 or 200 μg) of construct coding for Rad21. The variant allele of rs2839494 decreased estrogen receptor α (ERα) binding and E2-regulated activity. **(D)** Binding of 21q22.3 to Rad21 or ERα in MCF-7 cells was detected by chromatin IP. The *TFF1* promoter sequence (shown as *TFF1*), a well-known sequence to which ERα binds, served as the positive control. Real-time qPCR was performed to measure the amount of bound 21q22.3 fragment. The results are expressed as the change in threshold cycle (ΔCT). **(E)** Binding of the 21q22.3 fragment (that is, pGL3-803 bp) containing the rs2839494 wild-type (pGL3-803 bp (rs2839494 WT)) or variant allele (pGL3-803 bp (rs2839494 VT)) to ERα in MCF-7 cells is depicted. N.D, No data. **(F)** Graph depicts the regulatory activity on reporter of 21q22.3 containing the rs2839494 wild-type or variant allele in MCF-7 cells transfected with vector control (VC) or the Rad21-expressing construct (100 or 300 ng) in the presence or absence of 10 nM E2. **(G)** The hazard ratio (HR) of overall survival (OS) or disease-free survival (DFS) associated with SNP rs2251362 tagging the estrogen response element–associated region at 21q22.3 was significantly modified by the genotype of SNP rs959692 tagging the Rad21-containing block. In the subgroup showing a significant HR for OS (only in ER + patients), this HR remained significant. CI, Confidence interval.

On the basis of the ENCODE data, rs1078272 is located within the segment bound by Rad21, and the variant allele might affect the binding of Rad21 to the ERE-associated sequence at 21q22.3. This suggestion is supported by the finding that, in ChIP assays, the variant allele of rs1078272 led to decreased binding of the ERE-associated sequence to Rad21. An example is shown in Figure 6A, and the pooled results are shown in Figure 6B. Consistent with this hypothesis, as shown in Figure 6C, the dose-dependent increase in relative activity in the luciferase reporter assay caused by Rad21 (left panel) was abolished if this ERE-associated sequence contained the variant allele of rs1078272 (right panel).

It is possible that rs2839494 affected the binding of this sequence to ERα. This was confirmed by our findings that, in ChIP assays, in addition to binding to Rad21, this ERE-associated sequence at 21q22.3 could bind to ERα (Figure 6D) and that the variant allele of rs2839494 inhibited the binding of this sequence to ERα (Figure 6E). Furthermore, the increase in Rad21 coactivation of the ER-regulated activity caused by E2 was reduced when the wild-type allele of rs2839494 was replaced by the mutant allele (Figure 6F).

These findings prompted us to assess whether Rad21 plays a role in breast cancer progression, particularly via the mechanism proposed above. Interestingly, the SNP rs959692 tagging the *Rad21*-containing region was not itself associated with OS and DFS, but it did significantly modify the association between SNPs in 21q22.3 and breast cancer progression (Figure 6G). It is notable that, after further subgrouping the patients who showed a significant HR for OS, this HR remained significant in ER + patients, but not in ER - patients (see ER + and ER - in Figure 6G), a finding in accordance with the results of the cell line–based experiments.

## Discussion

In the present study, we employed two important approaches that make it unique from others. First, in contrast to most genetic and molecular epidemiological studies of breast cancer focused on cancer incidence, we addressed the importance of genetic polymorphisms in determining breast cancer progression. This issue is certainly of more translational relevance, and may add significant prognostic value to the currently used indicators for outcome prediction in breast cancer progression. Second, we employed a novel methodological approach. The move from candidate gene association studies to GWAS has made it possible to explore the etiological contribution of genetic variants throughout the whole genome without relying on an *a priori* hypothesis. As a result, many novel loci have been identified; the exploration of the genes within these loci should provide information about which genes and biologic pathways are associated with complex diseases. With only a very few notable exceptions, however, the number of detected causal variants directly responsible for individual GWAS associations remains small [28]. Most loci identified by GWAS require further fine-mapping, which usually takes a long time and a tremendous effort. In the present study, to provide a partial solution, we used a hybrid method consisting of candidate gene and genome-wide approaches. The well-defined roles of the ER during breast tumorigenesis make it mechanistically reasonable to assume that polymorphic genetic variants of EREs, central nodes in the ER pathway, might underlie the variations seen between patients in their susceptibility to breast cancer progression. This candidate mechanism lends critical support to the biological plausibility and tumorigenic relevance of our findings. In addition, our genotyping of SNPs on the basis of genome-wide detection of ERE-associated sequences [8]-[10] provided us a unique opportunity to examine real ERα-binding sites comprehensively. The successful identification of two ERE-associated SNPs at 21q22.3 using these combined methods suggests that this is a promising approach which will benefit from the increase in publicly available genomic and epigenomic data and bioinformatics platforms and thus will become more feasible. For example, the ENCODE project [26], in which regions of transcription, transcription factor binding, chromatin structure and histone modification were systematically mapped on a genome-wide scale, has generated valuable information that can be combined with SNP database data to address genetic susceptibility to cancer development and progression.

Our reporter gene assay and ChIP results show that the ERE-associated sequence at 21q22.3 has regulatory activity and that rs2839494 and rs1078272 in this region are able to affect the binding of this sequence to ERα and Rad21, respectively, resulting in a difference in ERα-activated expression of the reporter gene and suggesting that Rad21 promotes ERα-regulated transcription. At the functional level, this is biologically plausible and consistent with the finding that cohesin, a multisubunit protein complex containing Rad21 that is required for activation of transcription of *Myc* by E2, binds to ERα, upon which the complex binds to an ERE 70-kb upstream of *Myc*[27]. Even though at the molecular level, alternative models remain possible, one of which is that the binding of Rad21 to rs1078272 is through ERα already bound to this SNP or to the 21q22.3 ERE-associated sequence. However, the finding reported in ENCODE (shown in Figure 3) clearly demonstrates that ERα and Rad21 can only bind to specific and different segments within this 21q22.3 sequence and that ERα-binding segment does not cover rs1078272. Furthermore, we conducted an experiment to show that the effect contributed by the interaction between Rad21 and the rs1078272-containing sequence to promote expression does not occur indirectly via ERα. In this experiment, we used a shorter, 414-bp, rs1078272-containing sequence (that is, the 3' part of the original 803-bp sequence without containing any half-ERE site), and we observed the same result as that shown in Figure 6C, suggesting that the functional interaction between rs1078272 and Rad21 is independent of ERα. To confirm this hypothesis in further studies, researchers can explore the structure of ERα and Rad21 bound to DNA and examine whether these two SNPs are located at the protein–DNA interfaces, affecting protein–DNA interaction. Other mechanisms, such as higher-order chromatin–protein interactions, cannot be ruled out.

A more intriguing question is which genes are regulated by the Rad21-promoted, ERα-activated mechanism suggested by our findings. We comprehensively checked the region 1,000 kb 5' to 1,000 kb 3' of rs2839494. The results show that, of the 32 genes in this region (Additional file 1: Figure S1A), 14 have been reported to be involved in various tumorigenic mechanisms (Additional file 1: Figure S1B). Next, on the basis of published information (see, for example [9],[29],[30]), public data sets and the RT-qPCR results in the present study (Additional file 1: Figure S1C), we examined (1) whether expression of these genes has been detected in breast tumor or breast cancer cell lines, (2) whether the expression of these genes is putatively E2-dependent and (3) whether the proteins encoded by these genes have a function that is involved in tumor metastasis (Additional file 1: Figure S1B). On the basis of these criteria, among E2/ER-responsive genes, *TFF2*, *TFF3* and *TMPRSS3* are less likely to be targets of the 21q22.3 ERE-associated sequence, because the functions of the proteins encoded by these three genes, as well as clinical observations, suggest that these proteins play a role as metastasis promoters [31],[32]. This finding is contrary to what we observed in our patients’that the wild-type alleles of the 21q22.3 SNPs caused increased expression of a reporter gene and were associated with better cancer progression. Next, although it was found to promote migration and invasion in some cell line models [23], *TFF1* remains a possible target on the basis of the following evidence. *TFF1* expression is known to be upregulated by E2 [33] and has been shown to be an inhibitor of breast cancer metastasis in an animal model [23]. More importantly, the majority of the published clinical observations have shown that TFF1-positive primary breast tumors have a better outcome profile [23], consistent with our finding that the wild-type alleles of the SNPs were associated with a decreased risk of poor survival. Chromatin conformation studies (for example, chromosome conformation capture (3C) [34]) will help determine whether this 21q22.3 ERE interacts with its target genes and regulates expression by acting as a long-range regulator. Interestingly, with our preliminary results detected by 3C, we have identified a specific region within *TFF1* which can form a secondary structure with the 21q22.3 ERE-associated sequence. This interaction is more obvious in ER + breast cancer cells than in ER - cells and can be enhanced by the addition of E2 (Additional file 2: Figure S2). No such interaction was detected between the 21q22.3 ERE-associated sequence and other genes (for example, *TMPRSS3*) within this region (unpublished observation).

In the same way that the effect of individual SNPs on cancer incidence is small, the polymorphic alleles of ERE-associated SNPs at 21q22.3 predispose carriers to only a moderately increased risk of poor survival. Thus, the significance of such SNPs depends not only on the effect of each SNP alone but also on the interaction between functionally related alleles of individual SNPs. Our finding showing that the association between survival and the 21q22.3 SNPs was significantly modified by the SNP tagging *ESR1* and the SNP tagging the *Rad21*-containing block (Figures 4 and 6G) is in line with this suggestion. The observed interaction between ERE-associated SNPs and either the *ESR1* SNP or the ER status of the tumor also provides evidence for the breast tumorigenic relevance of these ERE-associated SNPs.

In the present study, we identified genetic variations at 21q22.3 as important factors in susceptibility to breast cancer progression. We attempted to address the possibility of false-positives and the effects of multiple testing by demonstrating a significant *P*-value in the permutation test. Furthermore, the two independent cohorts of patients yielded consistent results. Together with the functional experiments in the present study, these associations suggest that Rad21 promotes the effect of ERα in activating expression of E2-responsive genes, such as *TFF1*, which affects patients’ risk of poor survival. Expression quantitative trait locus–based analysis to examine if there is a link between 21q22.3 SNPs and expression of target genes in human populations is certainly warranted. More importantly, in our present study, we started with comprehensive genome-wide screening for ERE-associated loci that were significantly associated with survival status of the patient and demonstrated that some ERE-associated SNPs showed a significant association with survival of breast cancer patients (Figure 2). This suggests that these SNPs are not just important ones, but the most important ones, in determining susceptibility to breast cancer progression. As a result, in our ongoing study, on the basis of the individual contributions of these significant ERE-associated SNPs to breast cancer progression, we are attempting to generate a genetic risk score that can predict the DFS of our patients. Our preliminary results show that the inclusion of data for these significant ERE-associated SNPs significantly increases the area under the receiver operating characteristic curve. This finding might be critical in the development of new therapeutic and diagnostic approaches for breast cancer.

## Conclusions

The promise of personalized medicine, in which the associated risk and the course of diseases, as well as the efficacy of treatment protocols, may be predicted on the basis of a person’s genotype, must been tempered with caution. Nevertheless, validated molecular tests to assess the patient’s germline DNA already drive therapeutic decision-making [7]. On the basis of the well-documented role of ER in breast cancer progression, we explored whether genetic variations in EREs, the sequences bound by ER to activate the transcriptional regulation of target genes, are associated with breast cancer progression. Notably, the ERE sites genotyped have been shown to bind ERα *in vivo* using ChIP-based methods on a genome-wide scale, providing a unique opportunity to comprehensively examine putative ERE sites without depending on an *a priori* hypothesis. The SNPs at the 21q22.3 ERE were found to affect the binding of ER to ERE, leading to a difference in ER-regulated transcription, and to be significantly associated with OS and DFS. These findings support the idea that functional variants in the ERα-regulating sequence at 21q22.3 are important in determining breast cancer progression, as well as providing support for a role of ERE SNPs in breast cancer progression.

## Authors’ contributions

CYS participated in the generation of the study concept and in the study design and coordination, and drafted the manuscript. The following authors made substantial contributions to the analysis, experiments and interpretation of data for the work. CNH and PEW carried out ERE site searches, genotyping and data analysis. HWC, YLH, WCC and LYH performed functional assays. The following authors made substantial contributions to the acquisition of the data: HMH, MFH and JCY carried out the participant recruitment and interpretation of clinical information. All authors participated in drafting the manuscript and revising it critically for important intellectual content. All authors agree to be accountable for all aspects of the study to ensure that questions related to the accuracy or integrity of any part of the work are appropriately investigated and resolved. All authors read and approved the final manuscript.

## Additional files

## Electronic supplementary material

Additional file 1: Figure S1.: Genes within the region 1,000 kb 5' to 1,000 kb 3' of rs2839494 in 21q22.3. **(A)** Exons (vertical red lines) and untranslated regions (blue) of all the genes and untranslated mRNAs (green) in this region, 14 of which have been reported to be involved in various tumorigenic mechanisms **(B)**. **(C)** Estradiol (E2)/estrogen receptor (ER)–dependent expression of mRNAs for these genes detected by RT-qPCR in ER-positive (MCF-7) and ER-negative (MDA-MB-231) breast cancer cell lines. The results are normalized to those for α-actin mRNA. N.D., not done. *P <0.05 for differences between the conditions with E2 and without E2 in the same cell. (TIFF 14 MB)

Additional file 2: Figure S2.: Chromosome conformation capture (3C) suggests that the 21q22.3 ERE-associated sequence interacts with *TFF1* sequence. **(A)** Schematic diagrams of the 21q22.3 region containing *TFF1*, *TMPRSS3*, 21q22.3 SNPs and restriction enzyme (*Eco*RI) sites and the primers of quantitative PCR (qPCR) used in 3C. **(B)** Hypothesized model showing that the 21q22.3 ERE-associated sequence, after binding by the E2–ERα–p21 complex, forms a secondary structure with a specific region within *TFF1*. After restriction enzyme digestion and sequence linking, qPCR was performed using the forward and reverse primers (that is, F-primer and R-primer shown in the figure). **(C)** Relative DNA amounts detected by qPCR and the interaction between 21q22.3 ERE-associated sequence and *TFF1*, measured by amplified qPCR product, are more significant in ER-positive breast cancer cell lines (that is, MCF-7 and T47D), than in ER-negative cells (that is, MDA-MB-231, HS578T and MDA-MB-453) and can be enhanced by the addition of E2 (detected in T47D). (TIFF 18 MB)

Below are the links to the authors’ original submitted files for images.

Authors’ original file for figure 1

Authors’ original file for figure 2

Authors’ original file for figure 3

Authors’ original file for figure 4

Authors’ original file for figure 5

Authors’ original file for figure 6

## Abbreviations

aOR: Adjusted odds ratio
ChIP: Chromatin immunoprecipitation
CI: Confidence interval
DFS: Disease-free survival
ENCODE: Encyclopedia of DNA elements
ERE: Estrogen response element
ERα: Estrogen receptor α
GWAS: Genome-wide association studies
LD: Linkage disequilibrium
OS: Overall survival
SNP: Single-nucleotide polymorphism
