# Supplementary figures and images for: Functional variants at the 21q22.3 locus involved in breast cancer progression identified by screening of genome-wide estrogen response elements
Source: Breast Cancer Res. 2014 Oct 9;16:455. doi: 10.1186/s13058-014-0455-1 (PMC4303134; doi:10.1186/s13058-014-0455-1)

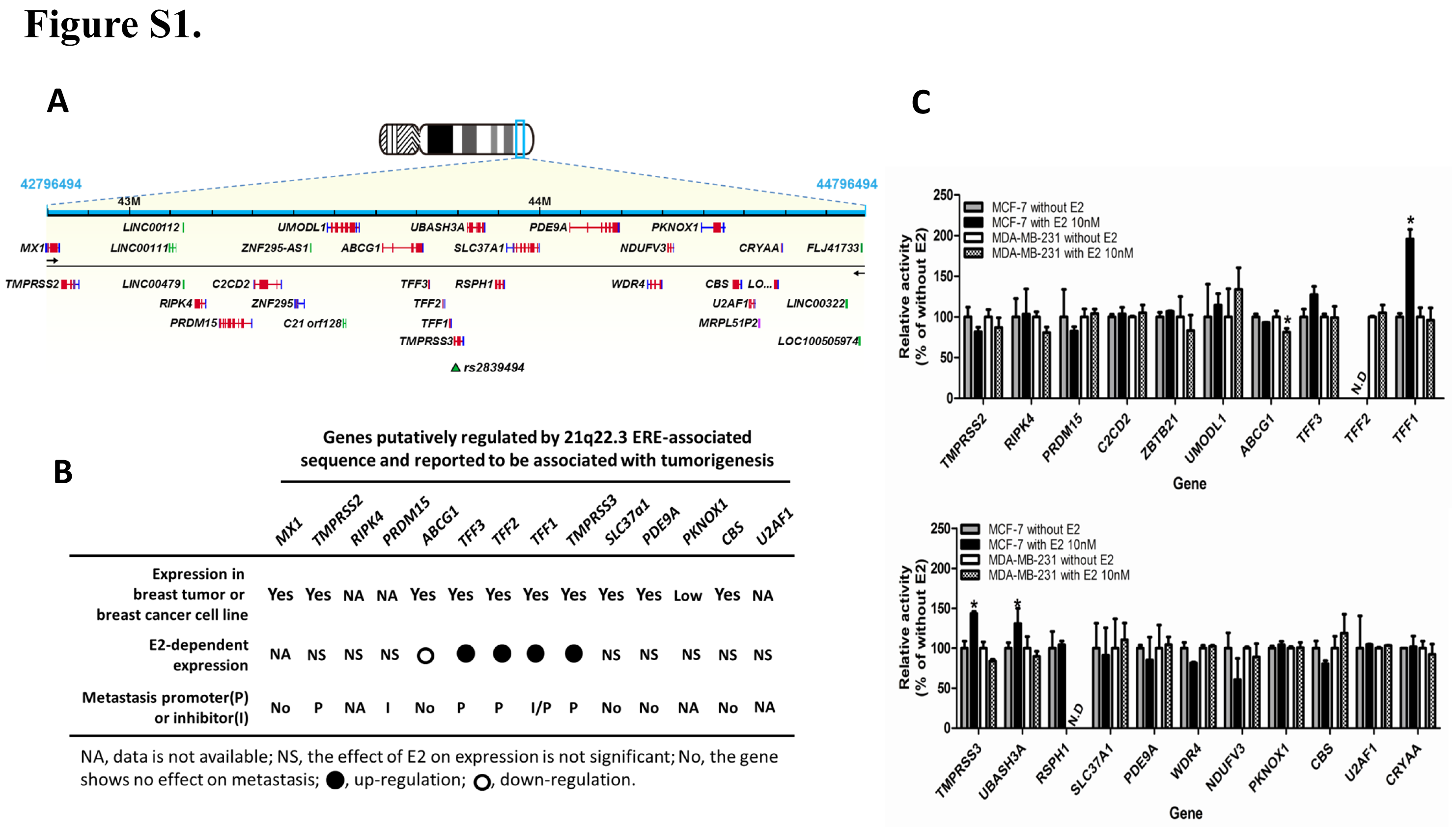

Supplement: Supplementary file 1 — Additional file 1: Figure S1.: Genes within the region 1,000 kb 5' to 1,000 kb 3' of rs2839494 in 21q22.3. (A) Exons (vertical red lines) and untranslated regions (blue) of all the genes and untranslated mRNAs (green) in this region, 14 of which have been reported to be involved in various tumorigenic mechanisms (B). (C) Estradiol (E2)/estrogen receptor (ER)–dependent expression of mRNAs for these genes detected by RT-qPCR in ER-positive (MCF-7) and ER-negative (MDA-MB-231) breast cancer cell lines. The results are normalized to those for α-actin mRNA. N.D., not done. *P <0.05 for differences between the conditions with E2 and without E2 in the same cell. (TIFF 14 MB) [file 13058_2014_455_MOESM1_ESM.tiff]

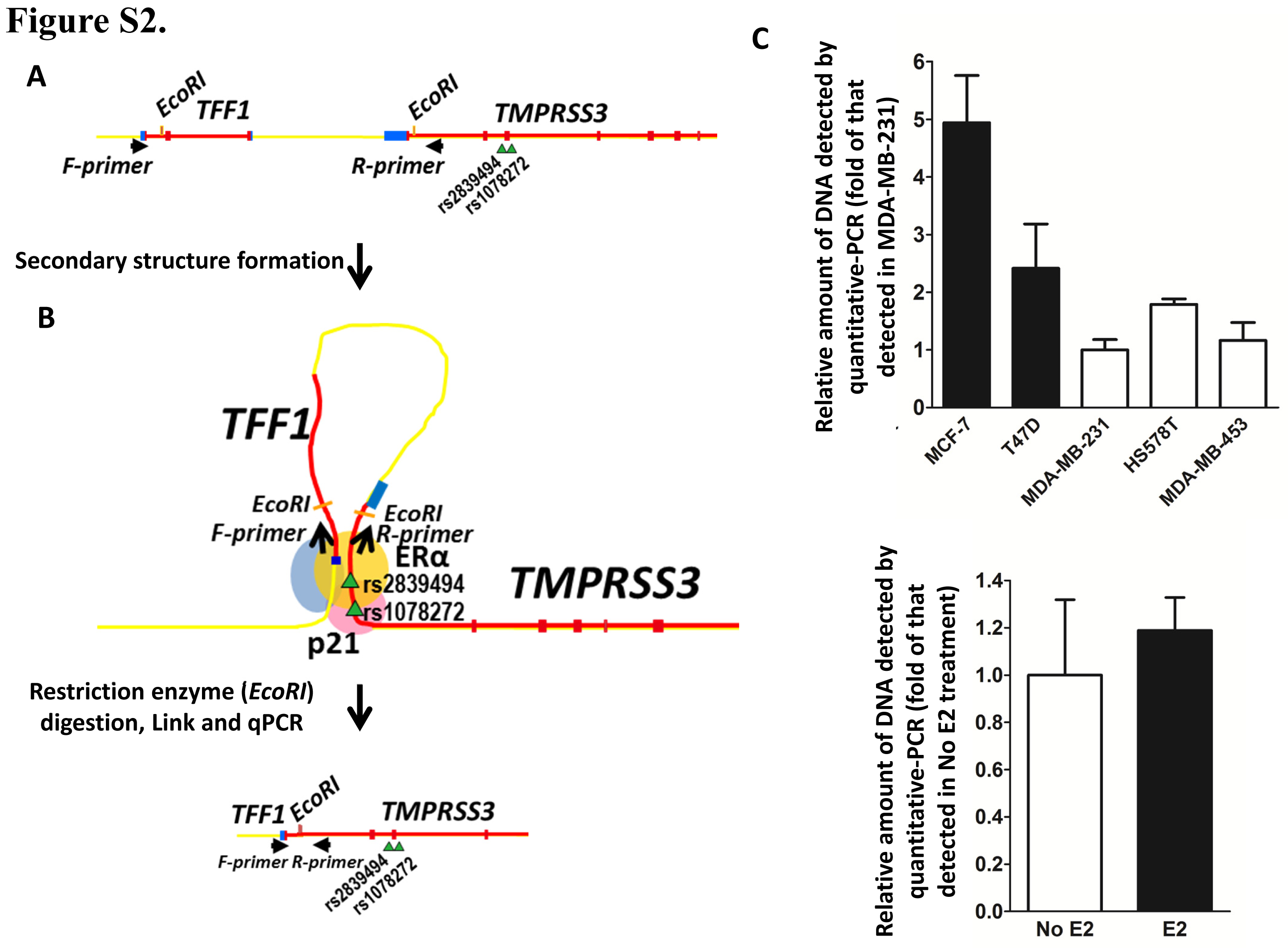

Supplement: Supplementary file 2 — Additional file 2: Figure S2.: Chromosome conformation capture (3C) suggests that the 21q22.3 ERE-associated sequence interacts with TFF1 sequence. (A) Schematic diagrams of the 21q22.3 region containing TFF1, TMPRSS3, 21q22.3 SNPs and restriction enzyme (EcoRI) sites and the primers of quantitative PCR (qPCR) used in 3C. (B) Hypothesized model showing that the 21q22.3 ERE-associated sequence, after binding by the E2–ERα–p21 complex, forms a secondary structure with a specific region within TFF1. After restriction enzyme digestion and sequence linking, qPCR was performed using the forward and reverse primers (that is, F-primer and R-primer shown in the figure). (C) Relative DNA amounts detected by qPCR and the interaction between 21q22.3 ERE-associated sequence and TFF1, measured by amplified qPCR product, are more significant in ER-positive breast cancer cell lines (that is, MCF-7 and T47D), than in ER-negative cells (that is, MDA-MB-231, HS578T and MDA-MB-453) and can be enhanced by the addition of E2 (detected in T47D). (TIFF 18 MB) [file 13058_2014_455_MOESM2_ESM.tiff]

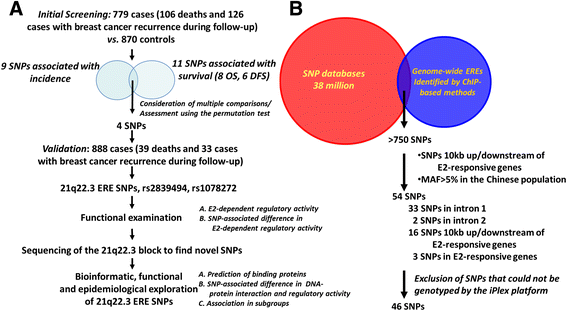

Supplement: Supplementary file 3 — Authors’ original file for figure 1 [file 13058_2014_455_MOESM3_ESM.gif]

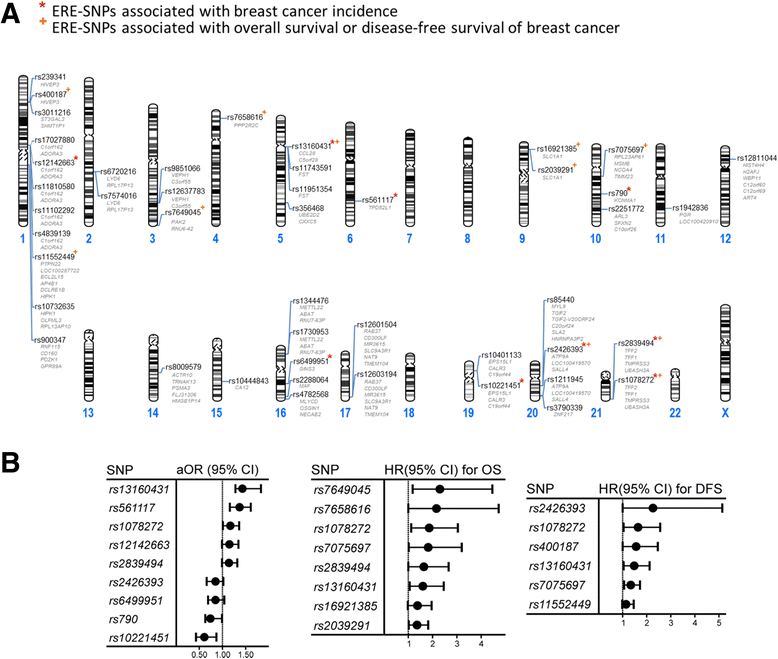

Supplement: Supplementary file 4 — Authors’ original file for figure 2 [file 13058_2014_455_MOESM4_ESM.gif]

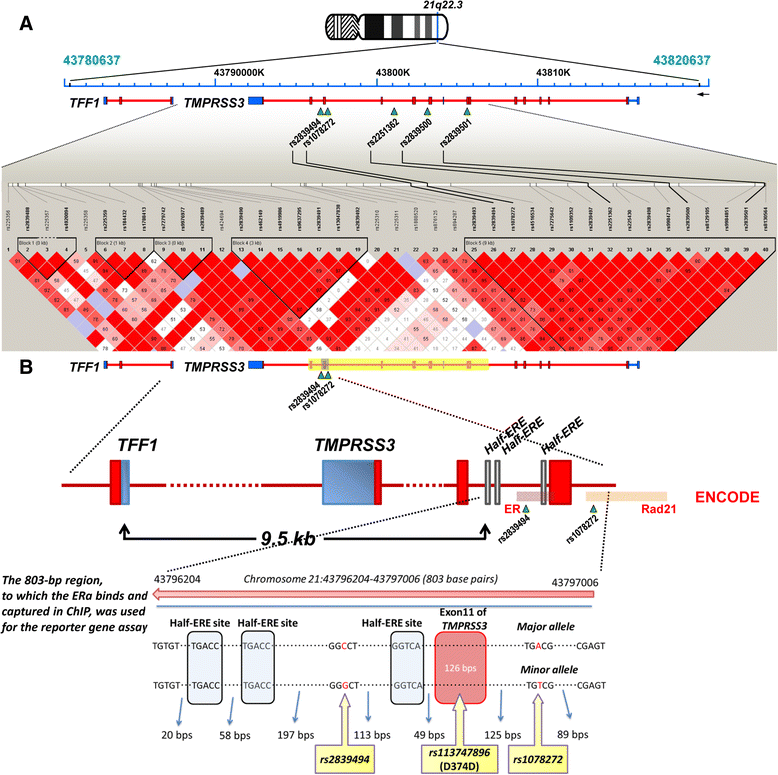

Supplement: Supplementary file 5 — Authors’ original file for figure 3 [file 13058_2014_455_MOESM5_ESM.gif]

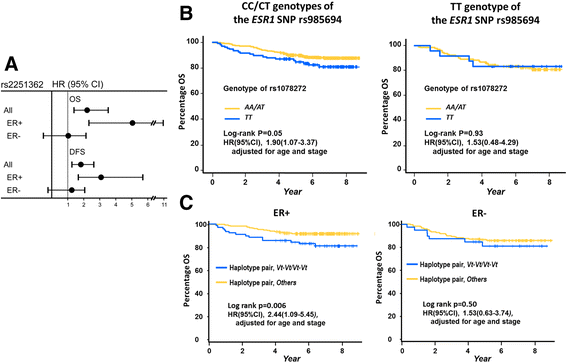

Supplement: Supplementary file 6 — Authors’ original file for figure 4 [file 13058_2014_455_MOESM6_ESM.gif]

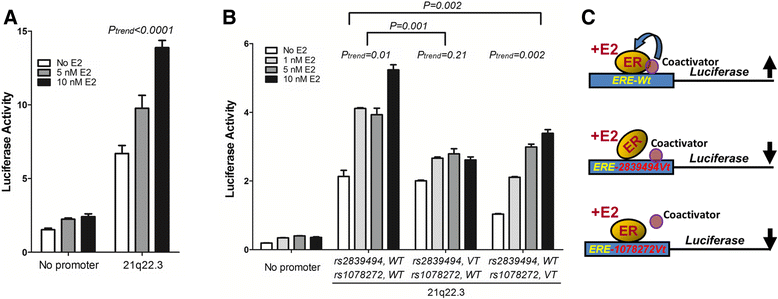

Supplement: Supplementary file 7 — Authors’ original file for figure 5 [file 13058_2014_455_MOESM7_ESM.gif]

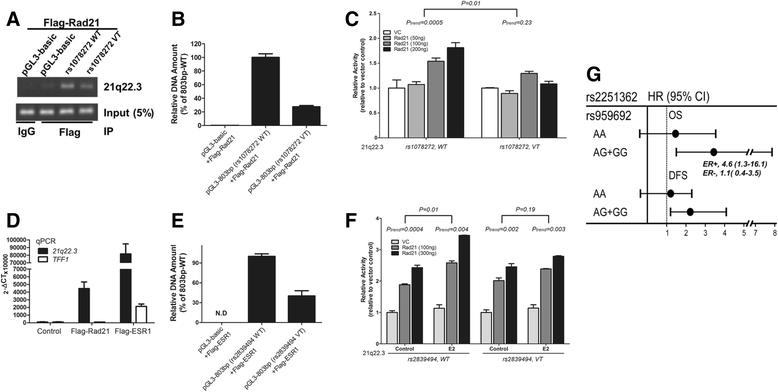

Supplement: Supplementary file 8 — Authors’ original file for figure 6 [file 13058_2014_455_MOESM8_ESM.gif]
